# Supplementary material for: Exploring the Therapeutic Potential of Ectoine in Duchenne Muscular Dystrophy: Comparison with Taurine, a Supplement with Known Beneficial Effects in the mdx Mouse
Source: Int J Mol Sci. 2022 Aug 24;23(17):9567. doi: 10.3390/ijms23179567 (PMC9455265; doi:10.3390/ijms23179567)
Supplement: Supplementary file 1 [file ijms-23-09567-s001.zip › ijms-1871385-SI.pptx]

## Slide 1
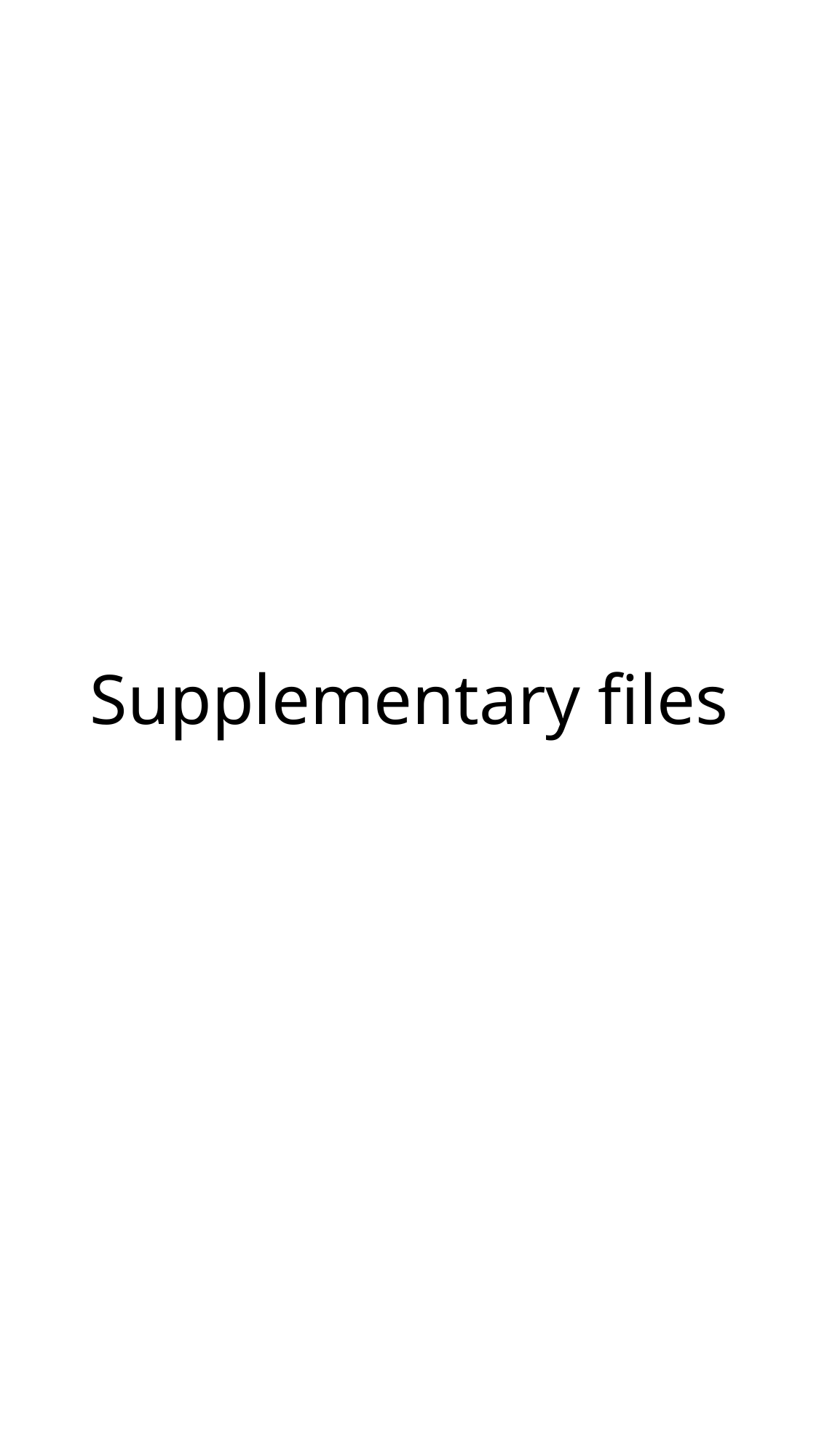

# Supplementary files

## Slide 2
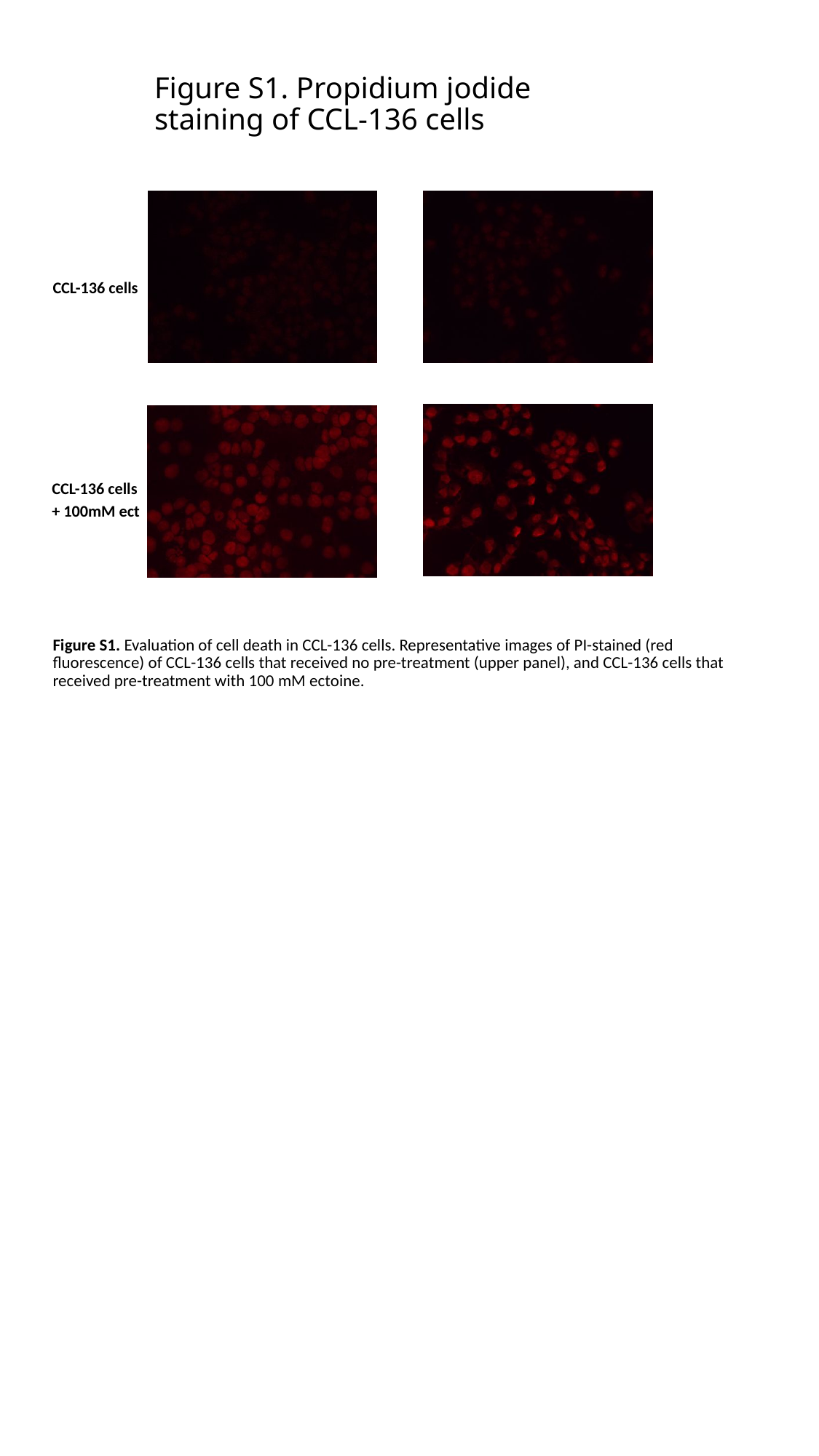

Figure S1. Propidium jodide staining of CCL-136 cells
CCL-136 cells
CCL-136 cells
+ 100mM ect
Figure S1. Evaluation of cell death in CCL-136 cells. Representative images of PI-stained (red fluorescence) of CCL-136 cells that received no pre-treatment (upper panel), and CCL-136 cells that received pre-treatment with 100 mM ectoine.

## Slide 3
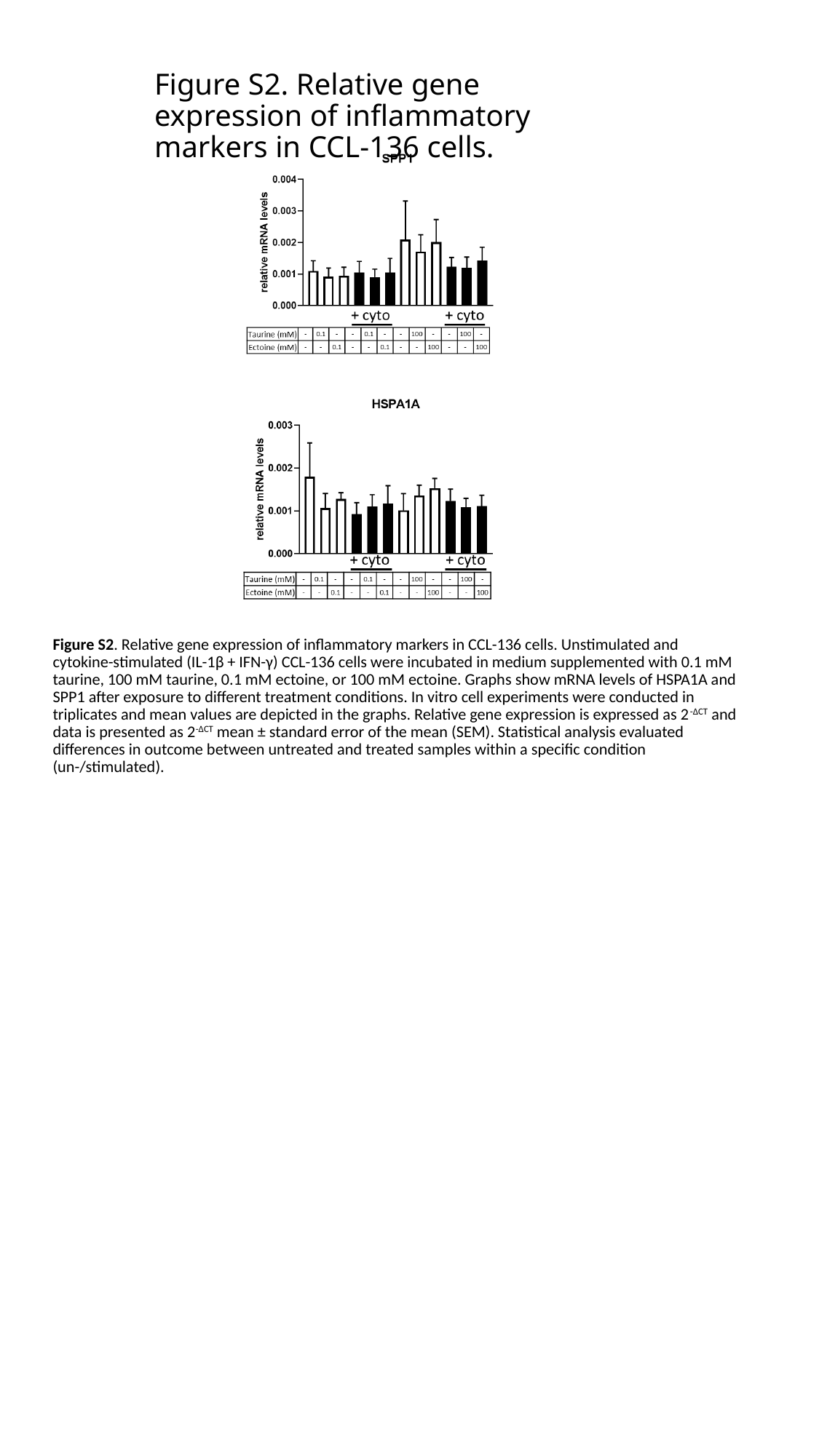

Figure S2. Relative gene expression of inflammatory markers in CCL-136 cells.
Figure S2. Relative gene expression of inflammatory markers in CCL-136 cells. Unstimulated and cytokine-stimulated (IL-1β + IFN-γ) CCL-136 cells were incubated in medium supplemented with 0.1 mM taurine, 100 mM taurine, 0.1 mM ectoine, or 100 mM ectoine. Graphs show mRNA levels of HSPA1A and SPP1 after exposure to different treatment conditions. In vitro cell experiments were conducted in triplicates and mean values are depicted in the graphs. Relative gene expression is expressed as 2-ΔCT and data is presented as 2-ΔCT mean ± standard error of the mean (SEM). Statistical analysis evaluated differences in outcome between untreated and treated samples within a specific condition (un-/stimulated).

## Slide 4
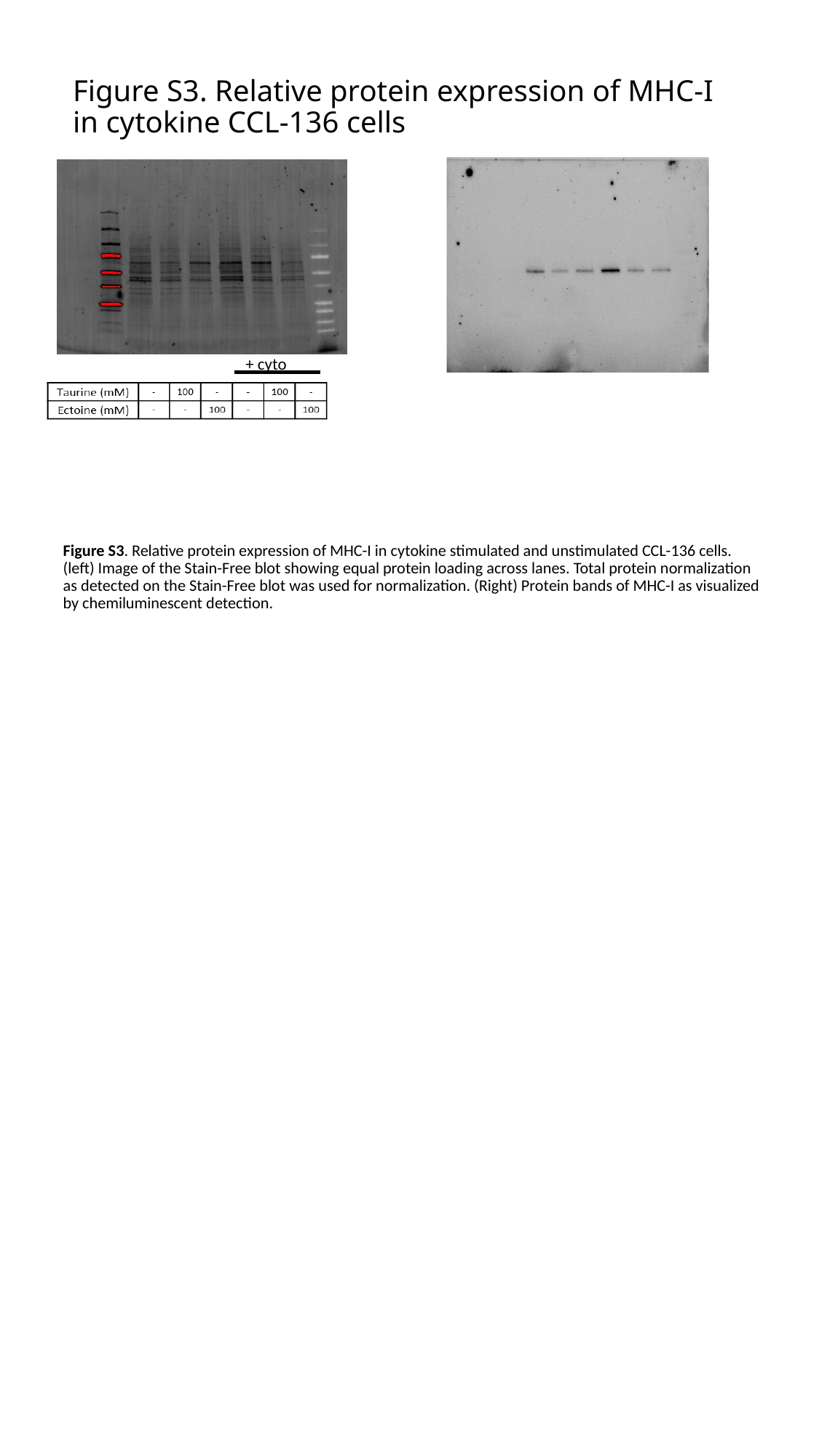

Figure S3. Relative protein expression of MHC-I in cytokine CCL-136 cells
+ cyto
Figure S3. Relative protein expression of MHC-I in cytokine stimulated and unstimulated CCL-136 cells. (left) Image of the Stain-Free blot showing equal protein loading across lanes. Total protein normalization as detected on the Stain-Free blot was used for normalization. (Right) Protein bands of MHC-I as visualized by chemiluminescent detection.

## Slide 5
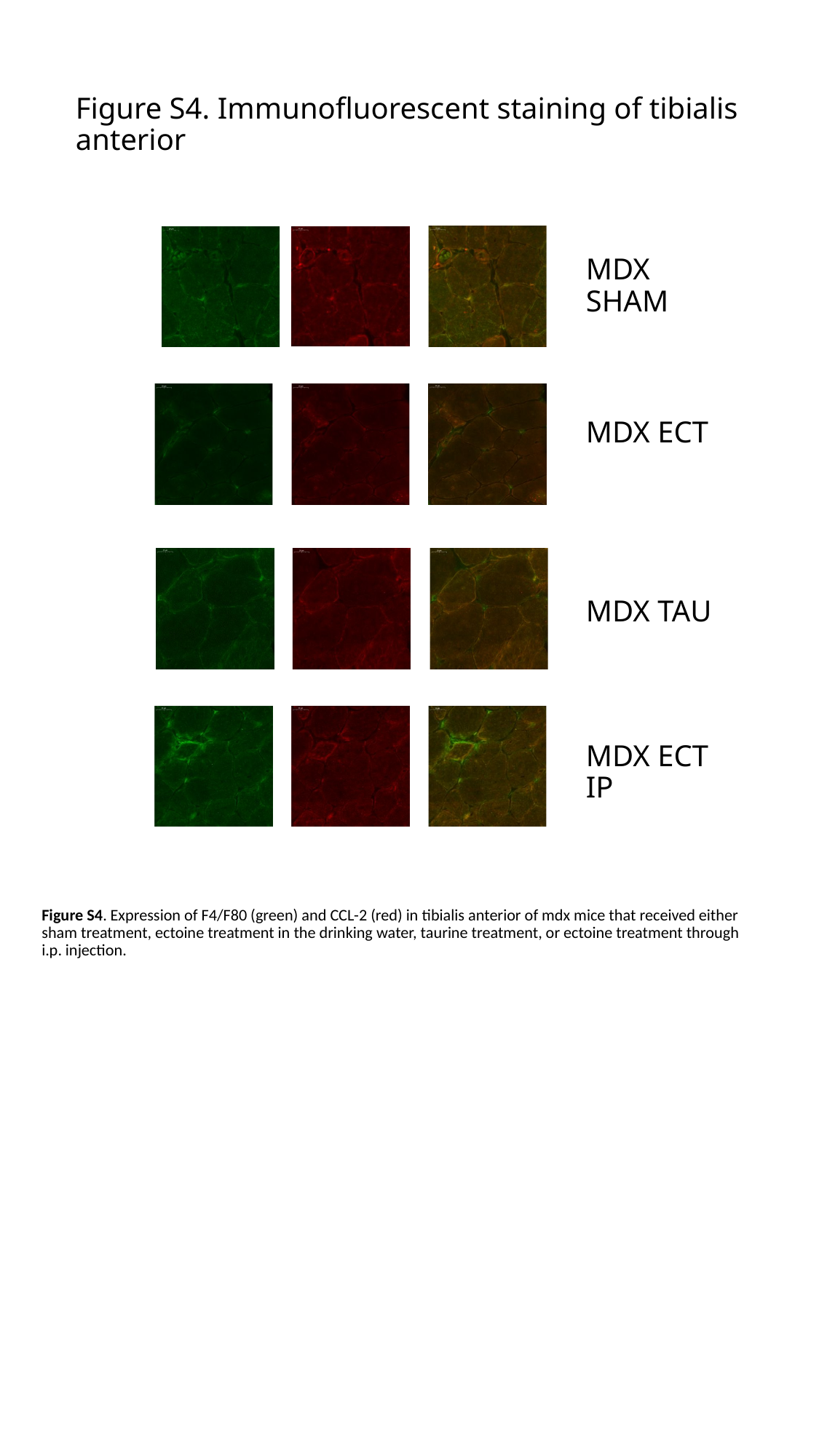

Figure S4. Immunofluorescent staining of tibialis anterior
MDX SHAM
MDX ECT
MDX TAU
MDX ECT IP
Figure S4. Expression of F4/F80 (green) and CCL-2 (red) in tibialis anterior of mdx mice that received either sham treatment, ectoine treatment in the drinking water, taurine treatment, or ectoine treatment through i.p. injection.

## Slide 6
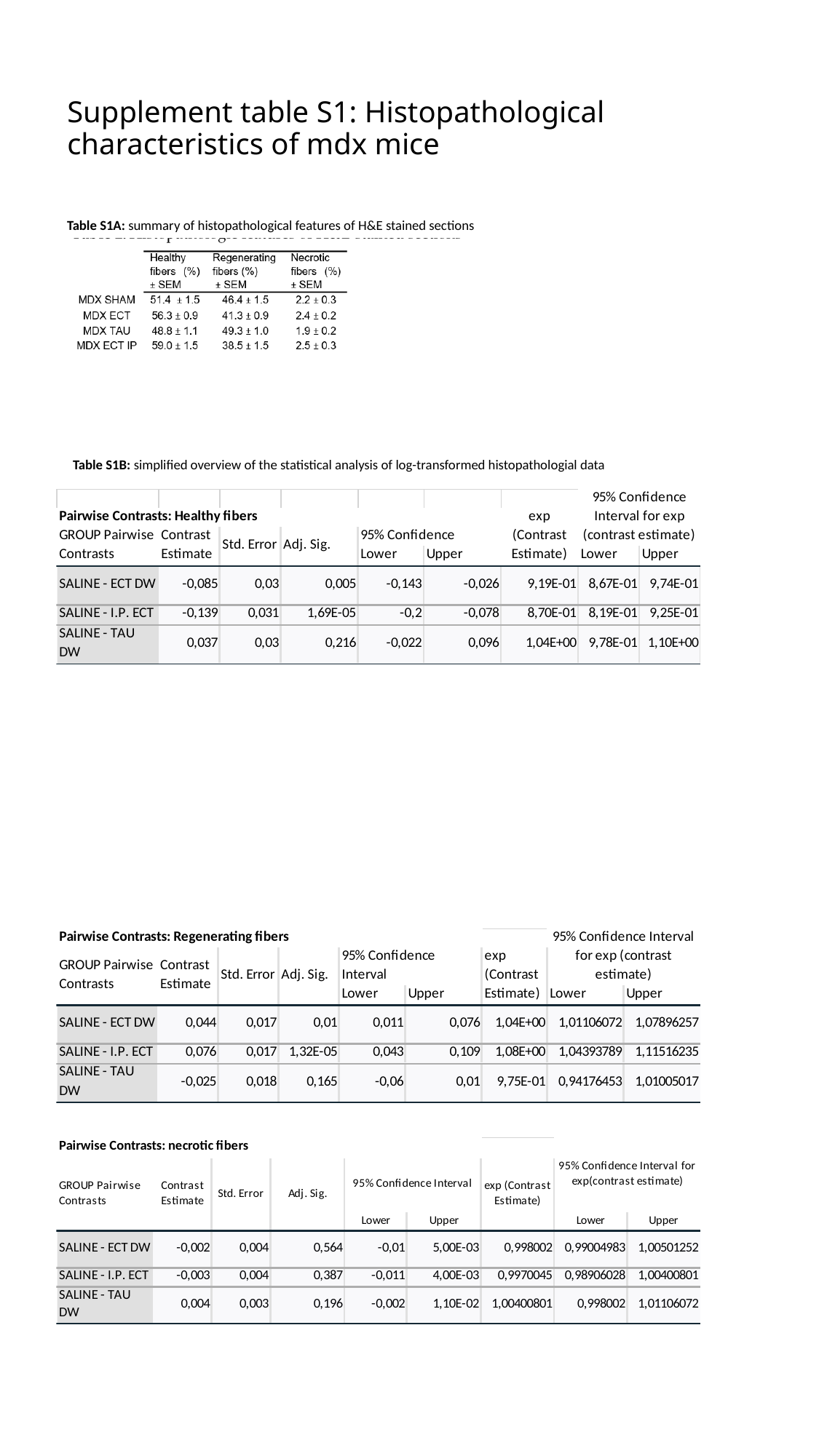

# Supplement table S1: Histopathological characteristics of mdx mice
Table S1A: summary of histopathological features of H&E stained sections
Table S1B: simplified overview of the statistical analysis of log-transformed histopathologial data

## Slide 7
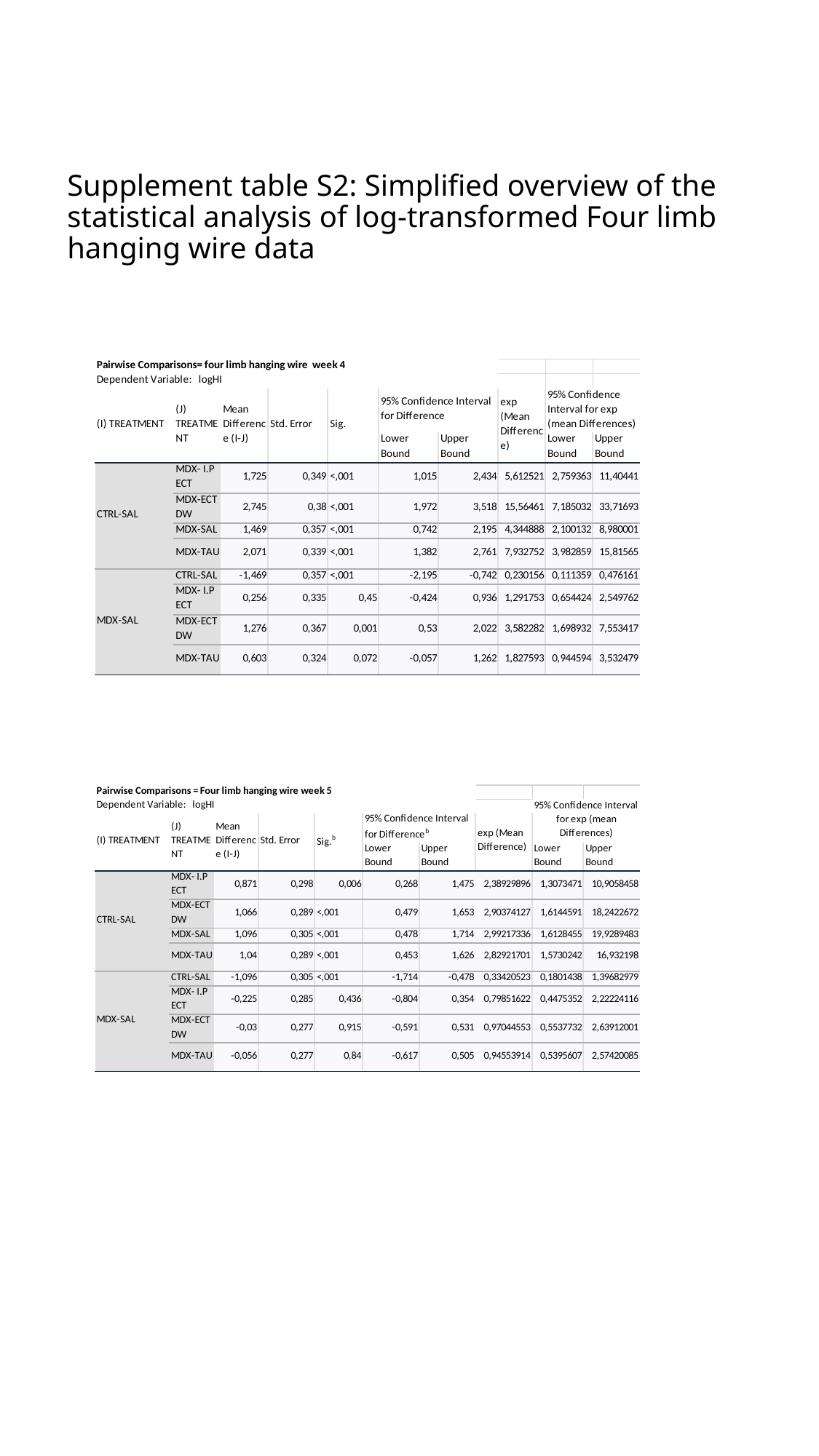

# Supplement table S2: Simplified overview of the statistical analysis of log-transformed Four limb hanging wire data

## Slide 8
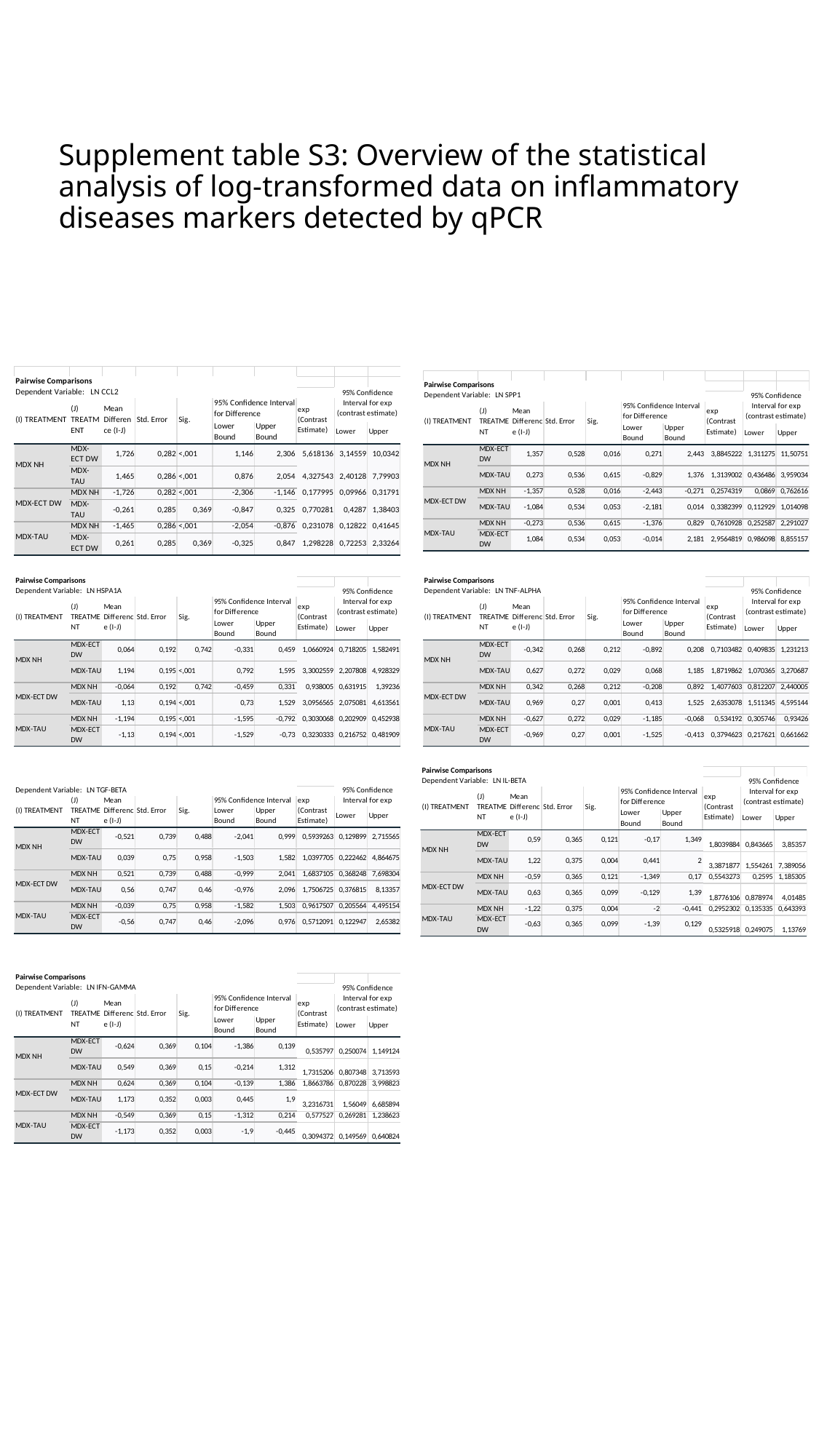

Supplement table S3: Overview of the statistical analysis of log-transformed data on inflammatory diseases markers detected by qPCR
